# Microsatellite marker development from next-generation sequencing in the New England cottontail (*Sylvilagus transitionalis*) and cross-amplification in the eastern cottontail (*S. floridanus*)

**DOI:** 10.1186/s13104-017-3062-2

**Published:** 2017-12-16

**Authors:** Timothy L. King, Michael Eackles, Aaron Aunins, Thomas J. McGreevy, Thomas P. Husband, Anthony Tur, Adrienne I. Kovach

**Affiliations:** 1 U.S. Geological Survey, Leetown Science Center, 11649 Leetown Road, Kearneysville, WV 25430 USA; 2 Natural Systems Analysts, Leetown Science Center, 11649 Leetown Road, Kearneysville, WV 25430 USA; 3 Department of Natural Resources Science, University of Rhode Island, 1 Greenhouse Road, Kingston, RI 02881 USA; 4 United States Fish and Wildlife Service, 300 Westgate Center Drive, Hadley, MA 01035 USA; 5 Department of Natural Resources and the Environment, University of New Hampshire, 56 College Road, Durham, NH 03824 USA

**Keywords:** Microsatellites, Cross-amplification, *Sylvilagus transitionalis*, *Sylvilagus floridanus*, *Sylvilagus obscurus*, Next-generation sequencing

## Abstract

**Objective:**

The New England cottontail (*Sylvilagus transitionalis*) is a species of high conservation priority in the Northeastern United States, and was a candidate for federal listing under the Endangered Species Act until a recent decision determined that conservation actions were sufficient to preclude listing. The aim of this study was to develop a suite of microsatellite loci to guide future research efforts such as the analysis of population genetic structure, genetic variation, dispersal, and genetic mark-recapture population estimation.

**Results:**

Thirty-five microsatellite markers containing tri- and tetranucleotide sequences were developed from shotgun genomic sequencing of tissue from *S. transitionalis*, *S. obscurus*, and *S. floridanus*. These loci were screened in *n* = 33 wild *S. transitionalis* sampled from a population in eastern Massachusetts, USA. Thirty-two of the 35 loci were polymorphic with 2–6 alleles, and observed heterozygosities of 0.06–0.82. All loci conformed to Hardy–Weinberg Equilibrium proportions and there was no evidence of linkage disequilibrium or null alleles. Primers for 33 of the 35 loci amplified DNA extracted from *n* = 6 eastern cottontail (*S. floridanus*) samples, of which nine revealed putative species-diagnostic alleles. These loci will provide a useful tool for conservation genetics investigations of *S. transitionalis* and a potential diagnostic species assay for differentiating sympatric eastern and New England cottontails.

## Introduction

The New England cottontail (*Sylvilagus transitionalis*) is a narrow niche specialist that relies on the dense understory vegetation of early successional or shrubland habitats [1]. These ephemeral habitats have declined steeply in recent decades in the Northeastern United States [2–4]. As a result, many shrubland species, including the New England cottontail, face severe population declines [5]. Remnant New England cottontail populations are found today in < 14% of the species’ historical range and occur in five geographically and genetically distinct populations located in southern Maine and southeastern New Hampshire, central New Hampshire, eastern Massachusetts on Cape Cod, eastern Connecticut and Rhode Island, and western Connecticut and New York [5]. Within these areas, cottontails face the consequences of population isolation and loss of genetic diversity [6, 7].

As a result of uncertainty in long-term species’ viability, the New England cottontail is a high priority for conservation in the Northeastern United States. To help recover the species, state and Federal natural resource managers collaborated with academicians and other stakeholders to develop and implement a conservation strategy to improve the outlook for the species [8]. As a result, the United States Fish and Wildlife Service determined listing of the species under the Endangered Species Act was no longer warranted [9]. Despite this decision, much uncertainty remains about the species’ viability and population status [7, 10]. To evaluate the effectiveness of the conservation strategy and inform adaptive modifications to improve outcomes, extensive population monitoring and research into population structure and genetic diversity are needed [8]. To this end, our goal was to develop a suite of microsatellite markers to aid future conservation genetics research on the New England cottontail as well as other *Sylvilagus* species.

## Main text

### Methods

For microsatellite marker development, total genomic DNA was extracted from tissue samples obtained from 13 New England cottontail, one eastern cottontail (*S. floridanus*), and two Appalachian cottontail (*S. obscurus*) using the DNEasy Blood and Tissue kit (Qiagen, Germantown, MD). We chose to sequence multiple species to maximize the number of microsatellites available for screening in the target species *S. transitionalis*. Sequencing libraries were created from these genomic DNA samples for sequencing on the Ion Torrent PGM and Ion Proton (ThermoFisher Scientific, Frederick, MD). For each sequencing library, the extracted DNA was quantified with a Nanodrop spectrophotometer (ThermoFisher Scientific), and 100 ng of DNA from each individual was used for construction of multiple 200 base pair libraries following the manufacturer’s protocol. For libraries with multiple individuals in one run, equimolar proportions of each library were pooled prior to chip loading.

Sequence reads from each completed run were imported into Qiagen CLC Genomics Workbench (ver. 7) for processing. Reads were length trimmed to a minimum size of 30 bp, and the quality trimming threshold was set to 0.01 corresponding to a Phred score of 20. Sequence reads from each of the 5 separate runs were then screened individually for all possible di-, tri-, tetra, penta-, and hexanucleotide microsatellite repeat motifs with a minimum repeat length of five with the program QDD (ver. 3) [11]. Primers were designed for each putative microsatellite locus within QDD using the integrated PRIMER 3 code [12]. Seventy-five tri- and tetra nucleotide loci identified by QDD with predicted amplified lengths between 100 and 250 bp were selected for screening within a collection of *n* = 8 wild-caught New England cottontails sampled from across the species range. A universal M13 sequence was added to the 5′ end of either the forward or reverse primer of each primer pair enabling an M13 FAM labeled fluorescent dye complementary to the universal tail to be incorporated into the polymerase chain reaction (PCR) product [13]. Polymerase chain reactions were performed in 25 μl volumes, consisting of 10 ng of DNA, 1 X PCR Buffer (Promega, Madison, WI), 0.25 μM of labeled forward primer, 0.5 μM of unlabeled reverse primer, 0.1 μM of labeled M13, 2.0 mM MgCl_2_, 0.2 mM of each dNTP, 0.25 units/μl Bovine Serum Albumin (New England Biolabs, Ipswich, MA), and 0.06 units/μl of Taq polymerase (Promega), using the following cycling conditions: 94 °C for 15 min, 29 cycles of 94 °C for 1 min, 58 °C for 45 s, and 72 °C for 45 s, 5 cycles of 94 °C for 1 min, 52 °C for 45 s, and 72 °C for 45 s, all followed by 72 °C for 10 min. PCR products for each locus were electrophoresed separately on an ABI 3130 Genetic Analyzer (ThermoFisher Scientific) automated DNA sequencer. Alleles were called using GeneMapper (ver. 4) (ThermoFisher Scientific) following the protocols described in [14].

Loci that amplified consistently and were easily scoreable on the sample of *n* = 8 wild New England cottontails were then tested on DNA extracted from an additional sample of *n* = 33 New England cottontails from a wild population in eastern Massachusetts, on Cape Cod. In addition, DNA extracted from a sample of *n* = 6 sympatric eastern cottontails, also collected from eastern Massachusetts were tested for cross-species amplification.

#### Data analyses

Genotype data from the Cape Cod New England cottontail collection (*n* = 33) were analyzed for null alleles, large allele dropout, and scoring errors using MICRO-CHECKER (ver 2.2.3) [15]. Genetic diversity and heterozygosity were quantified using GenAlEx 6.5 [16, 17]. Exact tests in GENEPOP [18] were used to determine if genotypes at each locus conformed to Hardy–Weinberg equilibrium (HWE). Multi-locus tests of conformance to HWE were completed using Fisher’s method in GENEPOP. Linkage disequilibrium (LD) was tested for all pairs of loci using contingency tables in GENEPOP. All tests of HWE and LD tests in GENEPOP used the default Markov chain parameters. Significance levels for HWE and LD tests were adjusted using the sequential Bonferroni correction [19].

To evaluate the utility of these loci for population genetic studies, multiple analyses were performed with the *n* = 33 New England cottontail collection. All multilocus genotypes were subjected to analysis via GENECAP [20] to identify matching samples, calculate match probabilities, and estimate the sibling probability of identity (*PI*_*sibs*_) [21]. To assess the randomness of the collection (e.g., to ensure the collection did not consist of a small number of families), we analyzed for the presence of full-sibling families using the program COLONY v2.0 [22]. Settings for COLONY analyses included the assumption of male and female polygamy, no per locus genotyping error information, no inbreeding, long run length with full likelihood analysis, high likelihood precision, no allele frequency updates, and no sibship prior. Individual New England cottontails were analyzed as offspring without assignment of individuals as candidate males (fathers) or females (mothers), as these data were not available. While the inference of family relationships is weakened in this situation with no sex, age, relationship information, and the assumption of polygamy for both sexes, COLONY is predicted to be more accurate than pairwise estimates of relationships [22]. Statistical significance of any full-sib relationships was assessed through the *P* values reported by COLONY. Within a sample of individuals taken at random (with respect to kin) from a population, the frequencies of full and half sib dyads can be used to estimate the current effective size (*N*_e_) of the population. Therefore, COLONY was also used to estimate *N*_e_ and associated 95% confidence interval utilizing the estimates from the sibship assignment full likelihood method. To estimate whether the *N*_e_ has remained constant (i.e., achieved mutation-drift equilibrium; see [23]), we conducted analyses with BOTTLENECK [24], implementing a two-phased model of mutation (5% IAM; 95% SMM; [25]). Statistical significance of the BOTTLENECK analyses was determined with a Wilcoxon signed-rank test performed by the software.

### Results and discussion

Within the 44,084,987 million sequence reads analyzed across five runs of the Ion Torrent PGM and Ion Proton platforms, 1,420,351 million were identified by QDD software as containing microsatellites using our chosen filtering criteria. Of the 75 loci selected from among these sequences for screening in the sample of *n* = 8 New England cottontails, all but three amplified consistently. An additional 24 loci were monomorphic. Of the remaining 48 loci, 17 had two alleles, 16 had three alleles, 10 had four alleles, three had five alleles, and two had seven alleles. From this set of loci, we selected the 35 most polymorphic, including all with three to seven alleles and a few with two alleles for additional testing in the wild collection of *n* = 33 New England Cottontails and *n* = 6 eastern cottontails from the same population to test for cross-specific amplification. The primer sequences of these 35 loci are provided in Table 1.

**Table 1:** Characteristics of 35 microsatellite loci in *n* = 33 *Sylvilagus transitionalis* and cross-amplification in *n* = 6 *S. floridanus*

Locus	Primer sequences	Repeat motif	Genbank accession number	Allele size range	A	A_e_	H_e_	H_o_	F	*P* value	Number of alleles in *S. floridanus*
StrQ2	F: *TGTCTGGCTTATGAGAAAGGG R: ACTGCGGGACTTCATACTGC	AGC	KX530819	129–153	5	3.0	0.68	0.61	0.09	0.107	4
StrQ7	F: *TCGCTCTCTGTCTCTGCCTT R: GCCTGCACTCTCTAGCTTGG	ACT	KX530820	116–122	2	1.6	0.39	0.39	− 0.03	1.000	5
StrQ8	F: *TCCTGCCTTTGAGAGCATTT R: TGGGTGATGCCTCACTTAAT	AGAT	KX530821	149–169	6	2.8	0.65	0.66	− 0.03	0.813	5
StrQ10	F: TGATAGGAAGGTAAGTCAGTGGG R: *TGCTCTCAAGAACCCAGGAG	AGAT	KX530822	120–132	4	2.0	0.50	0.47	0.04	0.184	0
StrQ11	F: *ATCGAATTTCGTCTGCATCC R: AAGGGTAGCTCTCAAACACCA	ATCC	KX530823	188–192	2	1.2	0.14	0.15	− 0.08	1.000	5
StrQ12	F: TGATTCGGAGTGGTTATGAAGA R: *ATTGCAATGCCCAACACTG	ATC	KX530824	132–135	2	1.1	0.12	0.12	− 0.07	1.000	6
StrQ15	F: *GATGGTGGATAAGATAGAGGACA R: TATGGCTGGACCACACTCTG	AGAT	KX530825	177–193	5	2.9	0.67	0.58	0.12	0.110	4
StrQ16	F: *CAGATTGACGTTGAGCAGA R: TGCTTGCCTCTTTCAAATAAA	AGAT	KX530826	144–152	3	2.4	0.58	0.64	− 0.11	0.677	4
StrQ18	F: *ACTCAGAGATAATGCACCAAA R: AAGGGTTAGATGGCAAGGGT	ACT	KX530827	215–221	3	2.4	0.59	0.58	0.01	0.173	6
StrQ19	F: ACCATCCATGACTGGTGAAA R: *GCACATGGGACATAGAAGGC	ATCC	KX530828	154–166	3	1.9	0.47	0.31	0.32	0.016	3
StrQ20	F: TGATTGCCCAGAGAATTAGTACC R: *AGGTCTTCAGACTCACTCTGGT	ATCC	KX530829	201–217	2	1.5	0.36	0.39	− 0.12	1.000	6
StrQ21	F: *TTCAATTTGCACCAAGAAGC R: CACAAGGTAGCATACTGTGGC	ATCC	KX530830	188–192	2	2.0	0.50	0.42	0.13	0.484	3
StrQ23	F: *AGCCTGCATCCTTTAGGG R: GCATAAAGGTTAATGAATAAGATTGC	AGAT	KX530831	207–211	2	1.1	0.06	0.06	− 0.03	1.000	3*
StrQ24	F: TGCGACTATTTCCAAAGCAT R: *TTGTTAGTTTCTCACAGTGCTGC	AGAT	KX530832	200–212	4	2.4	0.60	0.61	− 0.03	0.752	4
StrQ25	F* TGCATATTTGTCTTTCATTCTCTGA R: AACAATGACAAATTACACAGCCA	AGAT	KX530833	127–135	3	1.4	0.30	0.30	− 0.04	0.631	5*
StrQ26	F* CCTCTCTGAAACTATGCCTTTC R: CCTACAGCACTCCTGACTCGT	AAGG	KX530834	144–160	5	3.4	0.71	0.65	0.08	0.806	6
StrQ27	F:*TTCATCATCTTGTTATTTCATAGAGAA R: TGGAGCTCTTGGAGTCTAATGTG	AAGG	KX530835	142–154	4	2.7	0.63	0.64	− 0.02	0.623	4
StrQ28	F: *GGAAGAAAGGAAGAAAGGAAGA R: GGAGACTTGGATTGAATTCTGG	AAGG	KX530836	200	1	1.0	0.00	0.00	N/A	N/A	5
StrQ30	F:*CTTAATTGTAAGGAGGGTAGGAAA R: CCTCTTTCTCTCACTAATGCTGTT	AAGG	KX530837	102–138	6	3.8	0.75	0.73	0.02	0.436	0
StrQ31	F: *TCAAGCACAGAACAGAGCAG R: CCTTCTATTCCTTCTTTCTTCCTTC	AAGG	KX530838	130–134	2	2.0	0.50	0.46	0.08	0.726	5
StrQ32	F:*TGTAACTCTGCCTTCCAAATAAATAA R: GGCTGTTTAACTTGCCATGC	AAGG	KX530839	182–198	5	2.7	0.64	0.61	0.04	0.768	5
StrQ34	F:*TTAGAACGAACAAGAAAGAAAGAAA R: TCAAGTGGGAAAGGAATATCAA	AAGG	KX530840	148–156	3	2.5	0.62	0.58	0.05	0.415	4
StrQ37	F: *TTGTATGTGAACTTTGCACCG R: CACAGTCTAACTCTATCTGTCAAGAAA	AAGC	KX530841	94–106	2	1.5	0.34	0.24	0.28	0.123	6
StrQ39	F: *TCAAAGCACTTAATTCAAATCCC R: GTCTCTCCAAGCCTCCTCCT	AAGC	KX530842	192–196	2	1.9	0.48	0.33	0.29	0.135	2
StrQ41	F: *CCCTCTCTGTAACTCTGTCTTTCA R: TGAGGTACATTCAGCAGCCA	AAGG	KX530843	196–216	4	2.1	0.52	0.42	0.18	0.468	3*
StrQ42	F:*AGAAATAAAGAATAGCTTGGATAAATG R: CCTTCCTTCGTTCCTTCGTT	AAGG	KX530844	120–132	4	3.5	0.72	0.82	− 0.15	0.253	4*
StrQ43	F: *GTATCTGCCGAATGAAGTGGA R: TAGGTACAGATAATTCACTTTGGACA	AAGG	KX530845	218–230	3	1.9	0.49	0.55	− 0.13	0.637	3*
StrQ44	F:*TTATTTATTGGAAAGGTAGATAGGTGA R: TGGGTTATTGGCACTCACAA	AAGG	KX530846	230–234	2	1.3	0.26	0.24	0.06	0.551	4*
StrQ45	F: *CGACTAGGATAAATGCCACAGA R: CCCAATCCCTACACAACTAACAC	AAGG	KX530847	124	1	1.0	0.00	0.00	N/A	N/A	1
StrQ46	F:*AAATGAGGAATATTTGTTGAATGC R: AGAATGAACCAGCAAATGGG	AACT	KX530848	120–144	6	2.4	0.59	0.70	− 0.20	0.359	3
StrQ48	F: *GGGTTTGATGCTGAGGTCC R: TACATCCAGTGCACATTCGC	AACC	KX530849	118–122	2	1.1	0.06	0.06	− 0.03	1.000	2*
StrQ49	F: *ATCTTAGTGGAGAGAGTGGCTTG R: AAGTTCACCAGGCTAACTAGAGGA	AAAT	KX530850	120–132	4	3.0	0.68	0.58	0.14	0.178	1
StrQ63	F:*AAACAAGGAGATGTCACAGTAGTTTAG R: CATCCCTCTCTGTAACCTTGACTT	AATT	KX530851	126–130	2	1.3	0.24	0.21	0.10	0.467	2*
StrQ67	F: *TCTTGTCAAGTTTGTAACATGTGC R: TCAGCTTAACCCACTGTGCC	AATG	KX530852	246–254	3	1.3	0.22	0.24	− 0.12	1.000	2
StrQ72	F: *GCCTCTGCTTCTCTGTAACTTTG R: GGTTGGGAGGAGGCTTAGAG	AATC	KX530853	136	1	1.0	0.00	0.00	N/A	N/A	1

Locus refers to the name assigned to the microsatellite containing sequence. Primers sequences with a * denote which primer was tagged with an M13 tail. The number of alleles (A), effective number of alleles (A_e_), unbiased heterozygosity (H_e_), observed heterozygosity (H_o_), and fixation index (F) were output by the GenAlEx software. The Hardy–Weinberg *P* value is the *P* value reported across loci from Genepop using Fisher’s method. The Number of alleles in *S. floridanus* refers the number of alleles at each locus observed in *S. floridanus*, where a * denotes a putatively species specific amplification pattern not observed in *S. transitionalis*

Microchecker reported no instances of large allele dropout or scoring errors. Analyses of family structure in COLONY indicated the presence of no full sibling dyads, and therefore all individuals from the *n* = 33 collection were retained for subsequent analyses. Three loci were monomorphic in the Massachusetts wild New England cottontail population, while the remaining 32 loci averaged 3.1 alleles per locus (range 2–6; Table 1). Unique multilocus genotypes were generated for each individual with the probability of 6.4 × 10^−8^ that two siblings would share identical genotypes (*PI*_*sibs*_; [21]). Expected heterozygosity (H_e_) ranged widely across loci from 6.0% (StrQ23, StrQ48) to 75.0% (StrQ30) and averaged 47.0% for the Massachusetts wild New England cottontail population. Tests for conformance to HWE revealed that locus StrQ19 was not in HWE (*P* = 0.016), though this result did not remain significant after sequential Bonferroni correction. In addition, no statistically significant linkage disequilibrium (GENEPOP) was detected after sequential Bonferroni correction.

COLONY estimated the *N*_e_ (and 95% confidence limits) for the Massachusetts collection to be 48 (30–80), and BOTTLENECK indicated a statistically significant heterozygote excess for the Massachusetts collection (*P* < 0.001; Wilcoxon signed-rank test), suggesting a recent reduction in the effective population size. These results are consistent with previous work, showing that New England cottontails in eastern Massachusetts have low effective population sizes and have undergone a recent population decline [6, 26].

All but two loci amplified in the eastern cottontail samples, and an additional three loci were monomorphic (including two that were also monomorphic in New England cottontails). The remaining 30 loci had two to six alleles in eastern cottontail. Eight polymorphic loci (StrQ23, Str25, Str41, Str42, Str43, Str44, Str48, and Str63) had species-specific amplification patterns with no overlapping alleles across the screened individuals. In additional, StrQ72 was monomorphic for a different allele in each species. These results suggest a subset of these loci will be useful as a diagnostic screening tool for samples collected during noninvasive genetic monitoring surveys [10, 27]. However, more extensive testing of samples throughout the range where New England cottontail and eastern cottontail are sympatric is needed to confirm these results.

In conclusion, the markers developed in this study could prove useful for future population monitoring and conservation genetics research of New England cottontail. The high discriminatory power of these loci indicate that they will be particularly useful for noninvasive genetic surveys, where robust individual identification from a small panel of markers is required. Further, the cross amplification in eastern cottontails will facilitate use of these markers in this species as well as possibly others in the *Sylvilagus* genus.

## Limitations

These loci have been thoroughly screened in one of the geographically distinct populations of New England cottontails. Given effects of population isolation and genetic drift, it is likely that these markers may have different levels of polymorphism or null alleles in other geographic populations across the species range. Future work should screen the loci carefully in each geographic area prior to embarking on a new study. In addition, while we did not assess the genotyping error rate for these loci, future studies using them could measure this parameter to ensure accuracy of the genotype data.
